# The Synthetic Compound Norcantharidin Induced Apoptosis in Mantle Cell Lymphoma In Vivo and In Vitro through the PI3K-Akt-NF-**κ**B Signaling Pathway

**DOI:** 10.1155/2013/461487

**Published:** 2013-07-07

**Authors:** Hongyan Lv, Yan Li, Hengfei Du, Jie Fang, Xiaoning Song, Jinqiao Zhang

**Affiliations:** Department of Hematology, Third Hospital of Hebei Medical University, 139 Ziqiang Road, Shijiazhuang 050051, China

## Abstract

This study aimed to elucidate the antitumor activity of norcantharidin (NCTD) against human mantle cell lymphoma (MCL). Cell proliferation and apoptosis were examined by MTS and flow cytometry. Caspase-3, -8, and -9 activities were detected with a colorimetric caspase protease assay. Apoptotic proteins—including PARP, cyclin D1, Bcl-2 family proteins, XIAP, and cIAP I—were studied by western blot. The phosphoinositide 3 kinase (PI3K) inhibitor LY294002 was used to investigate the involvement of the PI3K/Akt signaling pathway. In vivo studies were performed using Z138 cell xenografts in nude mice. NCTD inhibited proliferation and induced apoptosis of Z138 and Mino cells, both in vitro and in vivo. PI3Kp110**α** and p-Akt expressions were downregulated by NCTD treatment. NCTD downregulated NF-**κ**B activity by preventing NF-**κ**B phosphorylation and nuclear translocation. This effect was correlated with the suppression of NF-**κ**B-regulated gene products, such as cyclin D1, BAX, survivin, Bcl-2, XIAP, and cIAP. This phenomenon was blocked by the PI3K inhibitor LY294002. Our results demonstrated that NCTD can induce growth arrest and apoptosis in MCL cells and that the mechanism may involve the PI3K/Akt/NF-**κ**B signaling pathway. NCTD may have therapeutic and/or adjuvant therapeutic applications in the treatment of MCL.

## 1. Introduction

 Mantle cell lymphoma (MCL) is a heterogeneous aggressive lymphoid malignancy that accounts for approximately 3–8% of non-Hodgkin's lymphoma (NHL) cases; it is characterized by the nonrandom chromosomal translocation t(11, 14) (q13; 32) and leads to overexpression of cyclin D1 [1]. There is currently no defined standard of care for patients with MCL. High-dose, multiagent chemotherapy induces high remission rates in previously untreated patients with MCL; however, relapse commonly occurs with a rather short median survival of 5 to 7 years [2, 3]. Clearly, novel agents and strategies are needed to improve clinical outcomes for patients with MCL [4].

 Cantharidin, the Chinese blister beetle, has anticarcinogenic properties. However, its severe urinary side effects limit its clinical application. Norcantharidin (NCTD), the demethylated form of cantharidin, has higher anticancer potentials, less nephrotoxicity, and fewer inflammatory side effects than cantharidin. NCTD also has the ability to increase white cells. Recent in vitro and in vivo studies have demonstrated that NCTD can inhibit tumor cell growth, including breast cancer, hepatoma, lung cancer, colorectal adenocarcinoma, and leukemic cells, while not harming normal hematopoietic cells [5–13]. 

 The antitumor mechanisms of NCTD are complex and poorly understood. Our previous study demonstrated that NCTD inhibits multiple myeloma cell growth and potentiates the antimyeloma effect of bortezomib by downregulating expression of the NF-*κ*B signal pathway [13]. In the present study, we evaluated both in vitro and in vivo anticancer activities of NCTD in MCL models with the aim of providing experimental therapeutic data to expand its indications in clinical trials.

## 2. Materials and Methods

### 2.1. Cell Culture and Reagents

 The human mantle cell lines Z138 and Mino were cultured in PRMI-1640 medium (Gibco BRL, Gaithersburg, MD, USA) containing 10% heat-inactivated fetal bovine serum (FBS; Gibco) in a humidified incubator with 5% CO_2_ at 37°C. NCTD (molecular weight 368.4, >99%) was purchased from Sigma Chemical Co. (St. Louis, MO, USA). Primary tumor cells were isolated from the peripheral blood of six MCL patients; all samples contained at least 85% CD19^+^/CD20^+^ B cells as detected by flow cytometry. Informed consent was obtained according to the Declaration of Helsinki, and the protocol was approved by the Third Hospital of Hebei Medical University Investigational Review Board.

 The annexin V staining kit was obtained from BD PharMingen (San Diego, CA, USA). Monoclonal antibodies against PARP, c-myc, cIAP1, XIAP, nuclear factor-*κ*B p65 (NF-*κ*B p65), the inhibitor of NF-*κ*B alpha (I*κ*B*α*), I*κ*B kinase (IKK*α*), and phosphorylated I*κ*B*α* (p-I*κ*B*α*), PI3Kp110*α*, Akt, and phospho-Akt (p-Akt) were obtained from Cell Signaling Technology (Danvers, MA, USA). Antibodies against Bcl-2, survivin, *β*-actin, and lamin B were from Santa Cruz Biotechnology (Santa Cruz, CA, USA). The Caspase Colorimetric Assay Kit was purchased from KeyGen Biotech Co., Ltd. (Nanjing, China). The phosphoinositide 3 kinase (PI3K) inhibitor LY294002 and the pan-caspase inhibitor carbobenzoxy-valyl-alanyl-aspartyl-[O-methyl]-fluoromethylketone (Z-VAD) were purchased from Cell Signaling (Boston, MA, USA) and BioVision Technology, Inc. (Mountain View, CA, USA), respectively.

### 2.2. Cell Viability and Apoptosis Assays

 The inhibitory effects of NCTD on MCL cell growth were determined by MTS with a CellTiter 96 AQueous Non-Radioactive Proliferation Assay (Promega). Z138 and Mino cells were treated with NCTD (0, 2.5, 5, 10, 20, or 40 *μ*M) for 24, 48, and 72 h. At the end of culturing, 20 *μ*L reagent was added to the culture medium and incubated for 3 h. The absorbance of formazan was measured at 490 nm. All experiments were performed in triplicate and repeated three times. The cell sensitivity to NCTD was expressed as the IC50 (50% inhibitory concentration).

Apoptosis was quantitatively determined by performing annexin V/PI staining following the manufacturer's directions. Cells were incubated with different concentration of NCTD (0, 2.5, 5, 10, 20, or 40 *μ*M) for 24 h. Approximately, 1 × 10^6^ cells were resuspended in 100 *μ*L binding buffer containing FITC-conjugated annexin V. PI was added, and cell analysis and data acquisition were performed using a flow cytometer (Beckman Coulter, Brea, CA, USA).

### 2.3. Spectrophotometric Detection of Caspase-3, -8, and -9 Activities

Cells were treated with different concentrations of NCTD (0, 2.5, 5, 10, 20, or 40 *μ*M) for 24 h or with 5 *μ*M NCTD for 12, 24, 36, and 48 h. Caspase-3, -8, and -9 activities were determined using the Caspase Colorimetric Assay Kit according to the manufacturer's instruction. Briefly, cell lysate (100 *μ*g total protein) was added to a reaction mixture, which contained colorimetric substrate peptides specific to caspase-3, -8, or -9. The reaction was incubated at 37°C for 4 h. A spectrophotometer was used to measure the absorbance at 405 nm, and the caspase activities were expressed as the ratio OD inducer/OD control. 

### 2.4. Cell Cycle Analysis

Cell cycle distribution was determined by staining DNA with PI (Sigma). Briefly, U266 cells were incubated with 0, 5, 10, and 20 *μ*M NCTD for 24 h. Approximately 1 × 10^6^ cells were collected and fixed in 70% ice-cold ethanol. Cell pellets were suspended in PI with simultaneous RNase treatment at 37°C for 30 min. Data acquisition and analysis were performed on a flow cytometer (Beckman Coulter).

### 2.5. Nuclear and Cytoplasmic Protein Fractionation

Z138 and Mino cells were treated with NCTD (0, 5, and 10 *μ*M) for 24 h. Nuclear and cytoplasmic proteins were extracted as previously described [13]. Cells were incubated in lysis buffer A (10 mM HEPES; 10 mM KCl; 0.1 mM EDTA, pH 6.8; 0.1 mM EGTA, pH 7.0; and 1.0 mM DTT) containing protease and phosphate inhibitors. Then, 10% NP-40 was added, the mixture was vortexed and microcentrifuged, and the supernatants were collected as cytoplasmic protein extracts. The pellets were resuspended in 25 mL buffer B (20 mM HEPES, pH 7.9; 400 mM NaCl; 1.0 mM EDTA, pH 8.0; 1.0 mM EGTA, pH 7.0; and 1.0 mM DTT) with ×1 protease and phosphate inhibitors, incubated for 30 min, and then centrifuged. The supernatants were collected as nuclear protein extracts.

### 2.6. Total Protein Extraction and Western Blot

Z138 and Mino cells were treated with NCTD (0, 5, and 10 *μ*M) for 24 h. Equal amounts of lysate (40 mg) were separated by sodium dodecyl sulfate polyacrylamide gel electrophoresis (SDS-PAGE) and then transferred onto a polyvinylidene fluoride (PVDF) membrane. The membrane was incubated with blocking buffer and then immunoblotted with primary antibodies overnight at 4°C. Then the membrane was incubated with IRDye infrared secondary antibodies for 1 h at room temperature. The membrane was visualized with the Odyssey Infrared Imaging System (Lincoln, NE, USA).

### 2.7. Quantitative Real-Time PCR (Q-PCR) and Semiquantitative RT-PCR

Z138 cells were treated with different concentrations of NCTD (0, 5, and 10 *μ*M) for 24 h and then harvested. Total RNA was extracted using TRIzol reagent according to the kit protocol (Invitrogen, Beijing, China). cDNA was reverse-transcribed using the PrimeScript RT Reagent Kit (TaKaRa, Dalian, China) according to the manufacturer's instructions. The Q-PCR reaction was performed following the kit protocol (TaKaRa Bio, Dalian, China), and amplification was performed using the ABI PRISM 7500 Real-Time PCR System (Applied Biosystems, CA, USA). The relative mRNA expression of each gene was normalized to GAPDH RNA levels and analyzed using the 2^−ΔΔCT^ method. The primers were synthesized by Sangon Bioteck (Shanghai, China). The primer sequences are shown in Table 1. 

For semiquantitative RT-PCR, PIK3CA and the housekeeping gene GAPDH were amplified using the primers listed in Table 1. PCR products were separated on 1.5% agarose gels and visualized. Images were captured with Image-Pro Plus Analysis Software (Media Cybernetics, Inc., USA) and used for densitometric analysis.

### 2.8. Animal Study

Z138 cells (10 × 10^6^ cells/animal) were injected subcutaneously into the subscapularis of 4-week-old male nu/nu nude mice (National Rodent Laboratory Animal Resource, Beijing Branch, China). The tumor volume (*V*) was calculated using the formula *V* = [tumor length in mm × (tumor width^2^ in mm)]/2. The xenografts of Z138 cells were grown to 100 mm^3^ and were treated with NCTD (20 mg/kg, IP, 3× per/week for four weeks) or normal saline (100 *μ*L, IP, 3× per/week for four weeks; *n* = 8/group). Tumor growth and animal body weights were monitored twice a week. Four hours after the last dose, mice were euthanized and the tumor xenografts were removed for immunohistochemical (IHC) and TUNEL analyses. The use of animals for research was approved by the Committee of Research Animals in the Third Hospital of Hebei Medical University.

### 2.9. TUNEL Assay

The TUNEL assay was used to detect apoptosis in tumor tissue (DeadEnd Colorimetric Apoptosis Detection System, Promega Biotech Co., Ltd.). Briefly, 5 *μ*m paraffin-embedded sections were treated with 20 *μ*g/mL proteinase K. DNA was end-labeled using Biotinylated Nucleotide Mix in TdT buffer at 37°C for 60 min. DNA strand breaks were visualized using DAB. Apoptotic cells were quantified by determining the percentage of apoptotic cells per field.

### 2.10. Immunohistochemical Analysis of NF-*κ*B

Immunohistochemical analysis of NF-*κ*B was performed as previously described [13]. Two independent pathologists who were blinded to the treatment groups reviewed the sections under a light microscope with ×40 power. Ten fields of interest were selected, and the percentage of immunohistochemically positive cells was determined for each selected field. The average percentage of positive cells for all fields was calculated and summarized as mean ± SD.

### 2.11. Statistical Analysis

The data were expressed as the mean ± SD and were analyzed using SPSS 16.0 software. Comparisons between groups were made using one-way or two-way ANOVA tests followed by Bonferroni post hoc tests. Differences were considered significant at *P* < 0.05. All statistical tests were two-sided. 

## 3. Results

### 3.1. NCTD-Induced Antiproliferative and Proapoptotic Effects in MCL Cells

**Figure 1:**
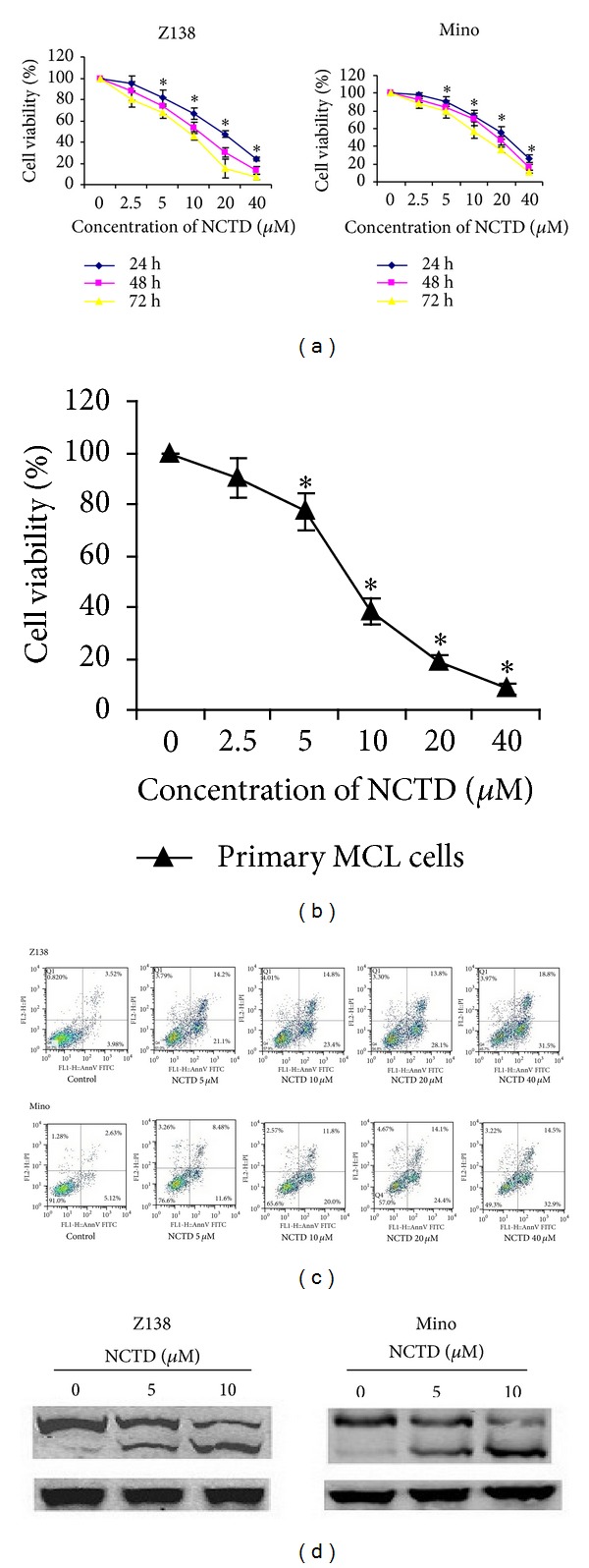
NCTD inhibited proliferation and induced apoptosis in MCL cells. (a) Z138 and Mino cells were treated with various concentrations of NCTD for 24, 48, and 72 h, and then cell viability was measured by MTS assay. (b) Primary MCL cells from six patients were treated with NCTD for 48 h and then by MTS assay. (c) Z138 and Mino cells were treated with different concentration of NCTD (0–20 *μ*M) for 24 h, and then apoptosis was detected using an annexin V/PI assay followed by flow cytometry. (d) PARP and cleaved PARP were determined by western blot. Experiments were performed in triplicate. Data are representative of three independent experiments. *P < 0.05 versus control.

MTS analysis showed that NCTD treatment resulted in significant time- and dose-dependent growth inhibition of both Z138 and Mino cells (Figure 1(a)). NCTD also induced dose-dependent cytotoxicity in primary MCL cells from six patients with de novo MCL (Figure 1(b)). Flow cytometry to detect NCTD-induced apoptosis revealed increased percentage of early and late apoptotic cells in both MCL cell lines (Figure 1(c)). Apoptosis was also confirmed by the appearance of PARP cleavage (Figure 1(d)).

### 3.2. NCTD-Induced Apoptosis Involves Caspase Activity

**Figure 2:**
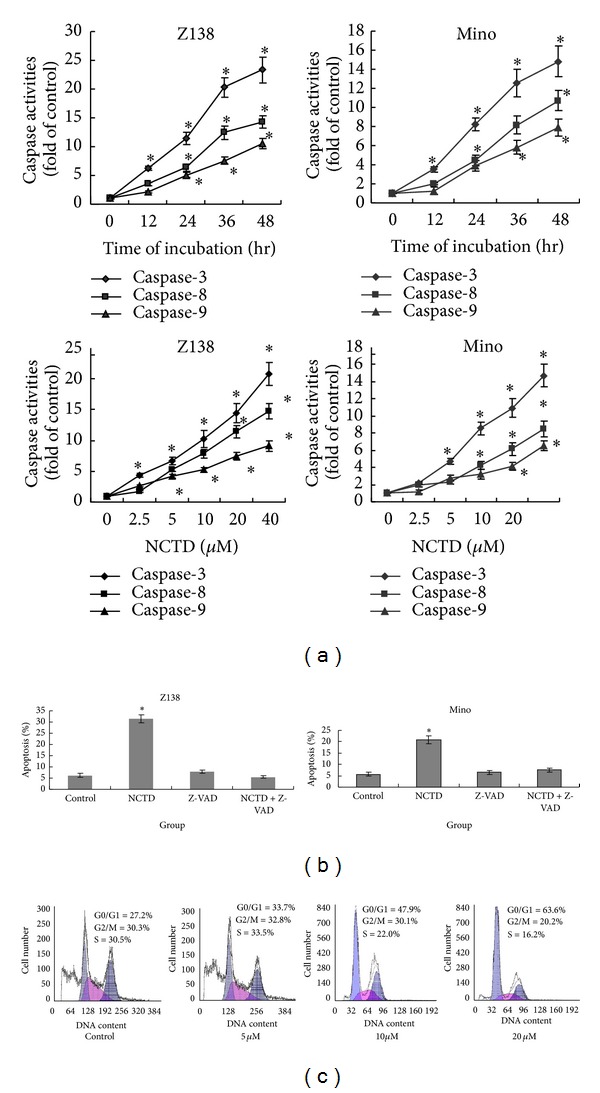
NCTD-induced caspase activities and G2/M arrest in MCL cells. (a) Z138 and Mino cells were treated with different concentrations of NCTD (0–40 *μ*M) for 24 h or with 5 *μ*M NCTD for 0, 12, 24, 36, and 48 h. Spectrophotometry was used to determine the activities of caspase-3, -8, and -9. (b) Z138 and Mino cells were preincubated with the pancaspase inhibitor Z-VAD-FMK (50 *μ*M) for 1 h before treatment with 5 *μ*M NCTD for 24 h, followed by the annexin V-PI assay. (c) Z138 cells were treated with different concentrations of NCTD (0, 5, 10, and 20 *μ*M) for 24 h, and then cell cycle analysis was performed using flow cytometry. Experiments were performed in triplicate. Data are represented as the mean ± SD (*n* = 3). *P < 0.05 versus control group.

To elucidate the molecular mechanisms by which NCTD induces apoptosis in MCL, we analyzed caspase action. NCTD treatment increased the activities of caspase-3, -8, and -9 in a time- and dose-dependent manner (Figure 2(a)). To determine whether NCTD-induced apoptosis was caspase dependent, the specific pan-caspase inhibitor Z-VAD (50 *μ*M) was added 1 h before the addition of 5 *μ*M NCTD. Flow cytometry analysis showed that Z-VAD completely abrogated NCTD-induced apoptosis (Figure 2(b)). 

### 3.3. MCL Cell Proliferation Is Inhibited due to G1 Arrest

To determine whether cell growth arrest was associated with NCTD treatment in MCL cells, cell cycle analyses were performed. NCTD treatment dose dependently resulted in an increased number of cells in the G1 phase of the cell cycle, with a concomitant reduction of cells in S phase (Figure 2(c)). 

### 3.4. NCTD Downregulated NF-*κ*B by Preventing NF-*κ*B Phosphorylation and Nuclear Translocation

**Figure 3:**
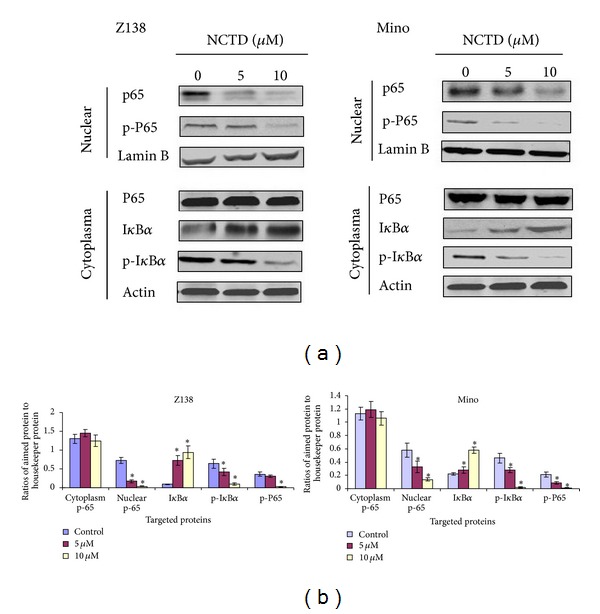
NCTD downregulated NF-*κ*B by preventing NF-*κ*B phosphorylation and nuclear translocation. Z138 and Mino cells were treated with 5 *μ*M NCTD for 24 h. (a) The protein expressions of NF-*κ*B p65, p-P65, I*κ*B*α*, and p-I*κ*B*α* were detected by western blot analysis. (b) Quantitative data of (a). Statistical analysis was carried out using the ANOVA and Bonferroni tests. *P < 0.05 versus control.

Previous studies have demonstrated that NF-*κ*B is constitutively active in MCL and that constitutive NF-*κ*B activation plays a key role in cell drug resistance MCL [14]. Therefore, we examined whether NCTD could reduce the constitutive NF-*κ*B activity in MCL cells. NCTD treatment downregulated the nuclear expressions of NF-*κ*B p65 and phosphorylated NF-*κ*B p65. The expression of NF-*κ*B p65 in cytoplasm was not affected by NCTD (Figures 3(a) and 3(b)). 

Nuclear translocation of NF-*κ*B occurs as a result of NF-*κ*B phosphorylation as well as I*κ*B*α* phosphorylation and degradation. Upon entering the nucleus, NF-*κ*B can bind DNA and cause gene transcription [15, 16]. We found that NCTD treatment inhibited I*κ*B*α* phosphorylation and increase total I*κ*B*α* in MCL cell cytoplasm (Figures 3(a) and 3(b)), thus inhibiting NF-*κ*B nuclear translocation. These results suggest that NCTD can sequester I*κ*Ba in the nucleus, resulting in the functional inactivation of NF-*κ*B. 

### 3.5. NCTD Also Downregulates NF-*κ*B-Regulated Gene Products

**Figure 4:**
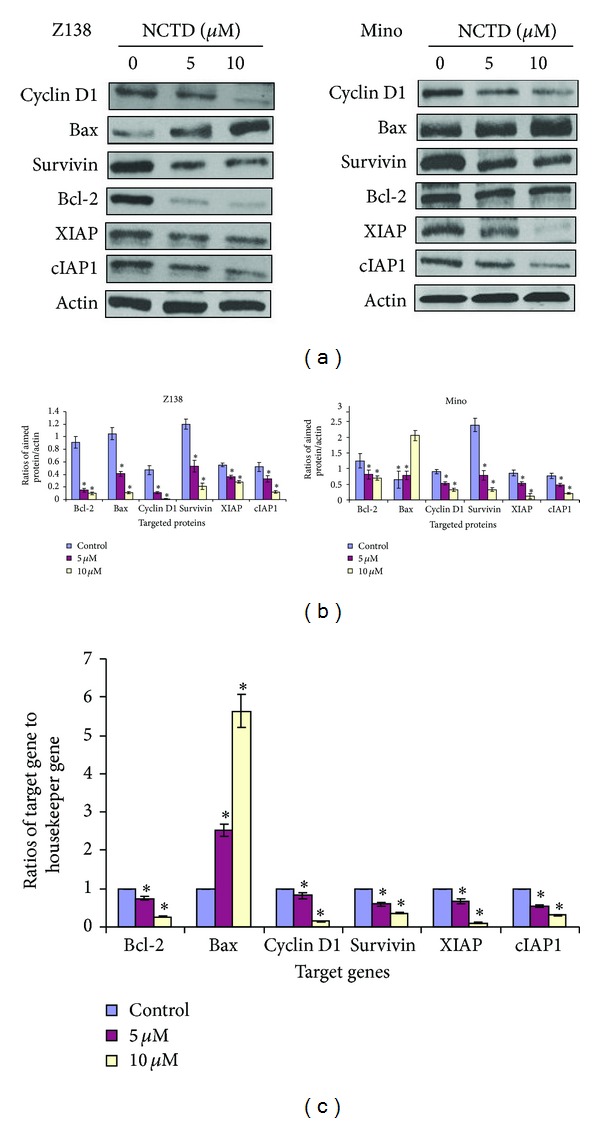
NF-*κ*B-regulated gene products were downregulated by NCTD. Z138 and Mino cells were treated with 0, 5, and 10 *μ*M NCTD for 24 h. (a) Western blot was used to analyze the expressions of Bcl-2, BAX, cyclin-D1, survivin, XIAP, and cIAP1. (b) Quantitative data of (a). (c) Z138 cells were treated with different concentrations of NCTD (0, 5, and 10 *μ*M) for 24 h, and then RT-qPCR was used to determine the mRNA expressions of Bcl-2, BAX, cyclin-D1, survivin, XIAP, and cIAP1. Statistical analysis was carried out using the ANOVA and Bonferroni tests. *P < 0.05 versus control.

Numerous proteins—including cyclin D1, BAX, survivin, Bcl-2, XIAP, and cIAP1—are regulated by NF-*κ*B and correlate with chemoresistance and apoptosis. We investigated whether the antilymphoma effects of NCTD were mediated by downregulated expression of NF-*κ*B-regulated products that are implicated in cell proliferation and apoptosis. Our results showed that NCTD downregulated the expressions of all of these molecules in protein level (Figures 4(a) and 4(b)) and gene level (Figure 4(c)). 

### 3.6. PI3K/Akt Survival Pathway Inactivation Is Associated with NCTD-Mediated Apoptosis

**Figure 5:**
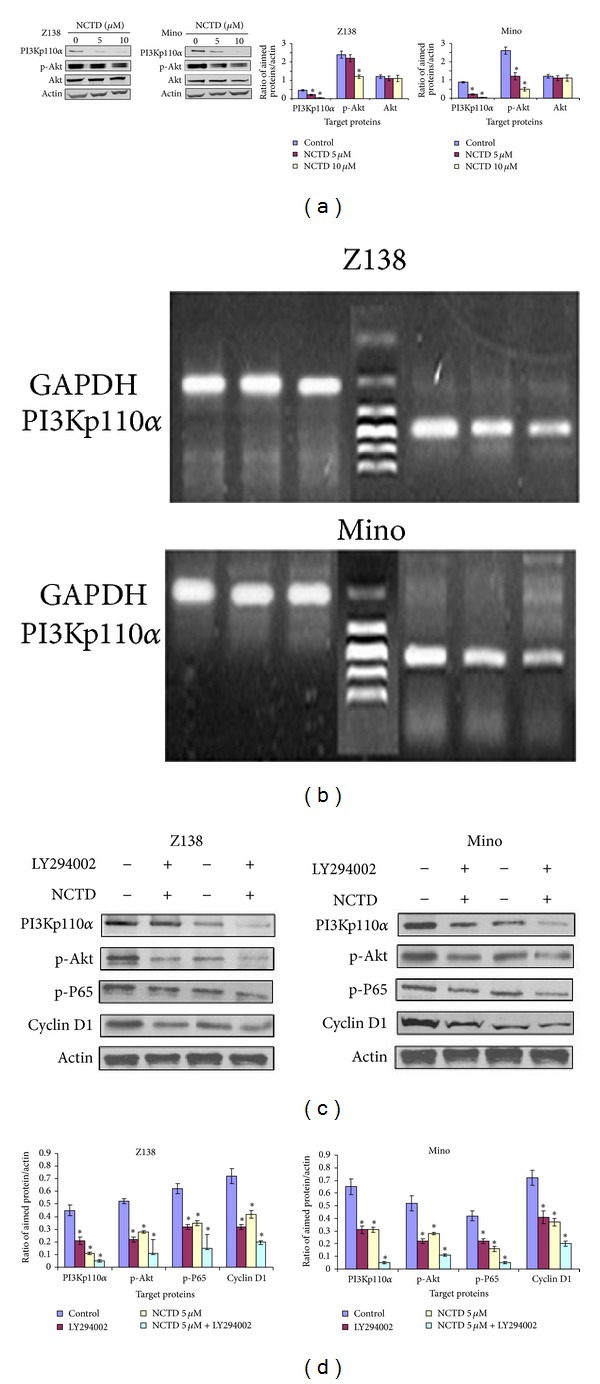
The PI3K/Akt survival pathway was associated with NCTD-mediated apoptosis. Z138 and Mino cells were treated with 5 *μ*M NCTD for 24 h. (a) Left, western blot was used to analyze the expressions of PI3Kp110*α*, p-Akt, and Akt. Right, quantitative data from these western blots for three independent experiments. (b) Inhibition of PI3Kp110*α* expression was confirmed at the mRNA level. (c) Z138 and Mino cells were preincubated with 50 *μ*mol/L LY294002 for 1 h before treatment of 5 *μ*M NCTD for 24 h. NCTD combined with LY294002 led to significant inhibition of the expression of PI3Kp110*α*, p-Akt, NF-*κ*B p-P65, and cyclin D1 proteins. (d) Quantitative data of (c). *P < 0.05 versus control.

We further investigated the PI3K/Akt survival pathway, which is known to affect the NF-*κ*B signal pathway, cyclin D1, and the IAP family. After treatment with NCTD for 24 h, there was a dose-dependent decrease in the levels of PI3Kp110*α* and p-Akt, while total Akt remained unchanged (Figure 5(a)). The time- and dose-dependent inhibition of PI3Kp110*α* expression after exposure to NCTD was further confirmed at the mRNA level (Figure 5(b)). 

To further verify the role of the PI3K/Akt pathway on NCTD-induced apoptosis, MCL cells were treated with NCTD in the presence or absence of the PI3K inhibitor LY294002 (50 *μ*mol/L). NCTD combined with LY294002 significantly inhibited the expression of p-Akt and therefore downregulated expression of p-P65 and cyclin D1 (Figures 5(c) and 5(d)).

### 3.7. NCTD Has Antilymphoma Effects In Vivo

**Figure 6:**
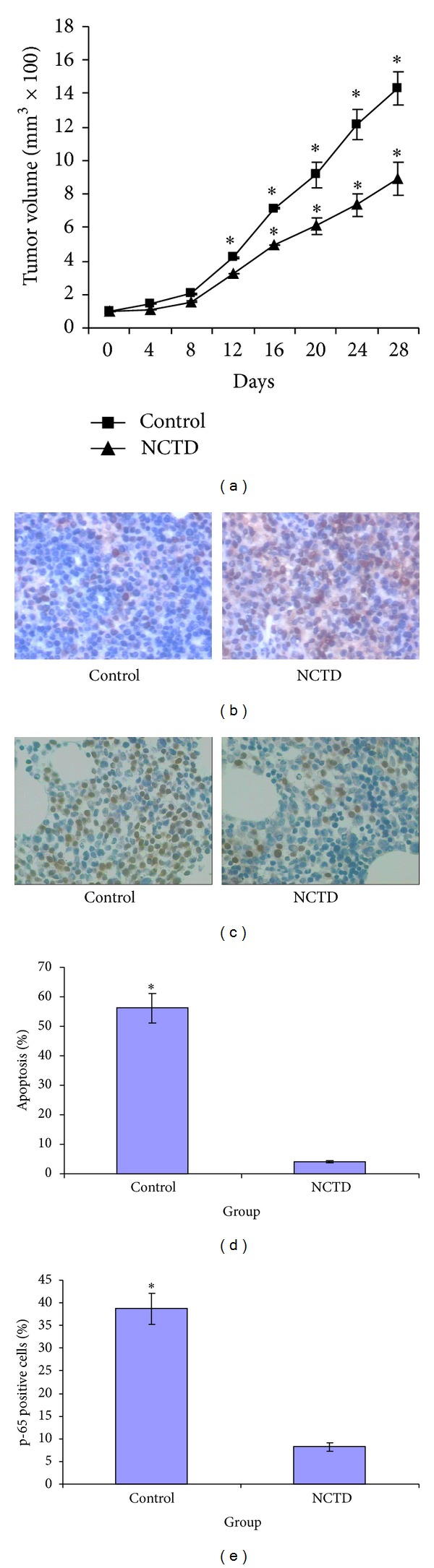
NCTD significantly inhibited Z138 nude mice xenografts. (a) Median tumor volumes of xenografts in mice receiving vehicle control or 20 mg/kg NCTD. Data represent mean ± SD of eight nude mice in each group. Statistical analysis was carried out performed using the ANOVA and Bonferroni tests. *P < 0.05 versus control. (b) Cell apoptosis determined by TUNEL method in MCL tumor samples. (c) Immunohistochemical analysis of NF-*κ*B expression in MCL mouse xenografts (400x). (d) Quantitative data of (b). (e) Quantitative data of (c). *P < 0.05 versus control.

Next, the antilymphoma effects of NCTD were evaluated in vivo. In an MCL-bearing mouse model established in nude mice, tumor growth was significantly inhibited by 20 mg/kg NCTD (*P* = 0.03, Figure 6(a)). With respect to the untreated control group, the tumor volume reduction rates were 37.4% in the NCTD group. Mouse body weight did not significantly change during the experiment. 

The TUNEL assay was used to detect apoptosis induced by NCTD. Our results revealed remarkably more frequent apoptosis in tumors harvested from the NCTD-treated animals than from the control group (*P* = 0.0011 and *P* = 0.0017; Figures 6(b) and 6(c)), demonstrating that NCTD effectively induced apoptosis in xenograft lymphoma tissues.

To verify the NCTD-mediated prevention of NF-*κ*B nuclear translocation in the animal model, we used IHC to detect NF-*κ*B p65 expression before and after NCTD treatment. Our data showed that NCTD treatment downregulated the nuclear expression of NF-*κ*B p65, suggesting that NCTD inhibited the nuclear translocation of NF-*κ*B.

## 4. Discussion 

The present study investigated the antitumor activity of NCTD against MCL. NCTD treatment resulted in dose-dependent cytotoxicity in Z138 and Mino cells as well as in primary MCL cells from six patients with de novo MCL. The anti-lymphoma effect of NCTD was confirmed in animal model also. Further experiments aimed to elucidate the mechanisms behind this effect.

MCL exhibits a complex pathobiology that includes cyclin D1 overexpression, abnormalities in the DNA damage response, and dysregulation of the PI3K/Akt and NF-*κ*B cell survival pathways [1, 17]. Activation of the PI3K/AKT pathway is a critical step in cell survival. AKT is a serine threonine kinase that regulates cell survival, proliferation, and apoptosis [1, 18–20], and constitutive AKT activation is reportedly essential to the pathogenesis and survival of MCL [21–23]. Previous in vitro testing of MCL cell lines with Akt inhibitors resulted in apoptosis via a caspase-dependent mechanism [23, 24]. Given the central role of the PI3K/AKT pathway in MCL, the present study examined the effects of NCTD on PI3K/Akt pathway regulation.

Akt is activated by phosphorylation, which is mediated by the direct binding of lipid second messengers, such as phosphatidylinositol-3,4-bisphosphate (Ptdins(3,4)P2) and phosphatidylinositol-3,4,5-trisphosphate (Ptdins(3,4,5)P3), which are generated by PI3K [25]. Akt activation can be induced by amplification of the PIK3CA gene, which has been implicated as a potential predictive oncogene in tumors [26]. Our present data showed that NCTD inhibited Akt activation through inhibition of PIK3CA gene transcription. The PI3K inhibitor LY294002 initiated the blockade of Akt signaling, thus inhibiting Akt phosphorylation. Combination with LY294002 potentiated the ability of NCTD to induce apoptosis of MCL cells and significantly downregulated Akt phosphorylation, indicating that inactivation of the PI3K/Akt survival pathway played an important role in NCTD-induced MCL cell death.

PI3K can regulate NF-*κ*B activation by the tyrosine phosphorylation-dependent pathway, thereby releasing NF-*κ*B to translocate to the nucleus [27]. Constitutive activation of NF-*κ*B is observed in MCL [1, 28, 29]. Here, we found that NCTD inhibited NF-*κ*B activity by blocking NF-*κ*B phosphorylation and nuclear translocation. When combined with LY294002, NCTD significantly affected NF-*κ*B P65 phosphorylation, confirming that NCTD negatively regulates NF-*κ*B activity through inactivation of the PI3K/Akt survival pathway. 

Cyclin D1 is overexpressed in MCL and is of paramount importance in determining the biological character of MCL. Recent studies show that NF-*κ*B transcriptionally regulates cyclin DI gene expression [30]. In our studies, we found that NCTD decreased cyclin D1 expression and that this inhibition was enhanced when NCTD was combined with LY294002. Thus, it is possible that NCTD prevents cyclin D1 expression by targeting the PI3K/Akt/NF-*κ*B signaling pathway.

Numerous proteins—such as survivin, XIAP, BAX, cIAP1, and Bcl-2—are regulated by NF-*κ*B and expressed in correlation with apoptosis and chemoresistance. Here we investigated whether the antitumor effects of NCTD were mediated by altered expressions of NF-*κ*B-regulated proteins that are implicated in cell proliferation and apoptosis. Our results showed that NCTD downregulated the expression of anti-apoptosis proteins (e.g., survivin, XIAP, cIAP1, and Bcl-2), and upregulated the expression of the proapoptosis protein BAX.

The anti-lymphoma effect of NCTD was also confirmed in a mouse model. NCTD treatment inhibited the growth of Z138 xenografts in nude mice, with concomitant decrease of the nuclear expression of NF-*κ*B p65. This finding further supports our hypothesis that inhibition of NF-*κ*B activity may be involved in the anti-lymphoma activity of NCTD in MCL.

## 5. Conclusion

The present preclinical study demonstrates the in vitro and in vivo antitumor activity of NCTD in MCL. NCTD induced marked antiproliferative and proapoptotic effects in MCL cells through modulation of the PI3K/Akt/NF-*κ*B pathway. These results support the rationale for clinical investigation of the potential therapeutic value of NCTD for MCL treatment.

## Figures and Tables

**Table 1:** List of primer sequences for RT-PCR.

Gene	Forward primer sequence	Reverse primer sequence	Size (bp)
Cyclin D1	5′-CTCCTGGTGAACAAGCTCAA-3′	5′-TGAACTTCACATCTGTGGCA-3′	160
XIAP	5′-AGCTTGCAAGAGCTGGATTT-3′	5′-ATTTGCACCCTGGATACCAT-3′	138
cIAP1	5′-AGAATTGGCAAGAGCTGGTT-3′	5′-CCGGTGTTCTGACATAGCAT-3′	118
Survivin	5′-CCTGGCAGCCCTTTCTCA-3′	5′-TCAGTGGGGCAGTGGATG-3′	121
Bcl-2	5′-AGTACCTGAACCGGCACCT-3′	5′-CAGCCAGGAGAAATCAAACA-3′	110
Bax	5′-TTGCTTCAGGGTTTCATCC-3′	5′-GACACTCGCTCAGCTTCTTG-3′	112
PIK3CA	5′-TGTGGGACTTATTGAGG-3′	5′-CACCATGATGTGCATCATTCA-3′	617
GAPDH	5′-GGGTGTGAACCATGAGAAGT-3′	5′-GGCATGGACTGTGGTCATGA-3′	143

## References

[1] Alinari L, Christian B, Baiocchi RA (2012). Novel targeted therapies for mantle cell lymphoma. *Oncotarget*.

[2] Herrmann A, Hoster E, Zwingers T (2009). Improvement of overall survival in advanced stage mantle cell lymphoma. *Journal of Clinical Oncology*.

[3] Martin P, Chadburn A, Christos P (2008). Intensive treatment strategies may not provide superior outcomes in mantle cell lymphoma: overall survival exceeding 7 years with standard therapies. *Annals of Oncology*.

[4] Williams ME, Dreyling M, Winter J, Muneer S, Leonard J (2010). Management of mantle cell lymphoma: key challenges and next steps. *Clinical Lymphoma, Myeloma and Leukemia*.

[5] Huang Y, Liu Q, Liu K, Yagasaki K, Zhang G (2009). Suppression of growth of highly-metastatic human breast cancer cells by norcantharidin and its mechanisms of action. *Cytotechnology*.

[6] Chang C, Zhu Y, Tang X, Tao W (2011). The anti-proliferative effects of norcantharidin on human HepG2 cells in cell culture. *Molecular Biology Reports*.

[7] Luan J, Duan H, Liu Q, Yagasaki K, Zhang G (2010). Inhibitory effects of norcantharidin against human lung cancer cell growth and migration. *Cytotechnology*.

[8] Chen Y-J, Chang W-M, Liu Y-W (2009). A small-molecule metastasis inhibitor, norcantharidin, downregulates matrix metalloproteinase-9 expression by inhibiting Sp1 transcriptional activity in colorectal cancer cells. *Chemico-Biological Interactions*.

[9] Chen Y-J, Kuo C-D, Tsai Y-M, Yu C-C, Wang G-S, Liao H-F (2008). Norcantharidin induces anoikis through Jun-N-terminal kinase activation in CT26 colorectal cancer cells. *Anti-Cancer Drugs*.

[10] Liao H-F, Su S-L, Chen Y-J, Chou C-H, Kuo C-D (2007). Norcantharidin preferentially induces apoptosis in human leukemic Jurkat cells without affecting viability of normal blood mononuclear cells. *Food and Chemical Toxicology*.

[11] Liu X-H, Blazsek I, Comisso M (1995). Effects of norcantharidin, a protein phosphatase type-2A inhibitor, on the growth of normal and malignant haemopoietic cells. *The European Journal of Cancer A*.

[12] Jiang YM, Meng ZZ, Yue GX (2012). Norcantharidin induces HL-60 cells apoptosis in vitro. *Evidence-Based Complementary and Alternative Medicine*.

[13] Du HF, Yu LJ, Meng YF (2013). Norcantharidin enhances bortezomib-antimyeloma activity in multiple myeloma cells in vitro and in nude mouse xenografts. *Leukemia Lymphoma*.

[14] Palumbo A, Attal M, Roussel M (2011). Shifts in the therapeutic paradigm for patients newly diagnosed with multiple myeloma: maintenance therapy and overall survival. *Clinical Cancer Research*.

[15] Baldwin AS (1996). The NF-kappa B and I kappa B proteins: new discoveries and insights. *Annual Review of Immunology*.

[16] Hideshima T, Chauhan D, Richardson P (2002). NF-*κ*B as a therapeutic target in multiple myeloma. *Journal of Biological Chemistry*.

[17] Leonard JP, Williams ME, Goy A (2009). Mantle cell lymphoma: biological insights and treatment advances. *Clinical Lymphoma & Myeloma*.

[18] Song G, Ouyang G, Bao S (2005). The activation of Akt/PKB signaling pathway and cell survival. *Journal of Cellular and Molecular Medicine*.

[19] Cantley LC (2002). The phosphoinositide 3-kinase pathway. *Science*.

[20] Fillmore GC, Wang Q, Carey MJ, Kim C-H, Elenitoba-Johnson KSJ, Lim MS (2005). Expression of Akt (protein kinase B) and its isoforms in malignant lymphomas. *Leukemia and Lymphoma*.

[21] Hofmann WK, de Vos S, Tsukasaki K (2001). Altered apoptosis pathways in mantle cell lymphoma detected by oligonucleotide microarray. *Blood*.

[22] Rizzatti EG, Falcão RP, Panepucci RA (2005). Gene expression profiling of mantle cell lymphoma cells reveals aberrant expression of genes from the PI3K-AKT, WNT and TGF*β* signalling pathways. *The British Journal of Haematology*.

[23] Rudelius M, Pittaluga S, Nishizuka S (2006). Constitutive activation of Akt contributes to the pathogenesis and survival of mantle cell lymphoma. *Blood*.

[24] Chappell WH, Steelman LS, Long JM (2011). Ras/Raf/MEK/ERK and PI3K/PTEN/Akt/mTOR inhibitors: rationale and importance to inhibiting these pathways in human health. *Oncotarget*.

[25] Kennedy SG, Wagner AJ, Conzen SD (1997). The PI 3-kinase/Akt signaling pathway delivers an anti-apoptotic signal. *Genes and Development*.

[26] Shayesteh L, Lu Y, Kuo WL (1999). PIK3CA is implicated as an oncogene in ovarian cancer. *Nature Genetics*.

[27] Béraud C, Henzel WJ, Baeuerle PA (1999). Involvement of regulatory and catalytic subunits of phosphoinositide 3-kinase in NF-kappaB activation. *Proceedings of the National Academy of Sciences of the United States of America*.

[28] Pérez-Galán P, Dreyling M, Wiestner A (2011). Mantle cell lymphoma: biology, pathogenesis, and the molecular basis of treatment in the genomic era. *Blood*.

[29] Pham LV, Tamayo AT, Yoshimura LC, Lo P, Ford RJ (2003). Inhibition of constitutive NF-*κ*B activation in mantle cell lymphoma B cells leads to induction of cell cycle arrest and apoptosis. *Journal of Immunology*.

[30] Witzel I-I, Koh LF, Perkins ND (2010). Regulation of cyclin D1 gene expression. *Biochemical Society Transactions*.

